# Update on Incidence, Prevalence, Treatment and Survival of Patients with Small Bowel Neuroendocrine Neoplasms in the Netherlands

**DOI:** 10.1007/s00268-021-06119-y

**Published:** 2021-04-24

**Authors:** Enes Kaçmaz, Arantza Farina Sarasqueta, Susanne van Eeden, Koen M. A. Dreijerink, Heinz-Josef Klümpen, Pieter J. Tanis, Els J. M. Nieveen van Dijkum, Anton F. Engelsman

**Affiliations:** 1 Department of Surgery, Amsterdam UMC, University of Amsterdam, Amsterdam, The Netherlands; 2 Amsterdam Center for Endocrine and Neuroendocrine Tumours (ACcENT), Cancer Center Amsterdam, ENETS Center of Excellence, Amsterdam, The Netherlands; 3 Department of Pathology, Amsterdam UMC, University of Amsterdam, Amsterdam, The Netherlands; 4 Department of Endocrinology, Amsterdam UMC, Vrije Universiteit Amsterdam, Amsterdam, The Netherlands; 5 Department of Medical Oncology, Amsterdam UMC, University of Amsterdam, Amsterdam, The Netherlands; 6 Department of Surgery, Cancer Center Amsterdam, Amsterdam UMC, Vrije Universiteit Amsterdam, de Boelelaan 1117, 1081HV Amsterdam, The Netherlands

## Abstract

**Background:**

Small bowel neuroendocrine neoplasms (SB-NEN) are rare cancers, population-based studies are needed to study this rare indolent disease. The aim of this study was to explore trends in epidemiology, treatment and survival outcomes of patients with SB-NEN based on Dutch nationwide data.

**Patients and methods:**

Patients with grade 1 or 2 SB-NEN diagnosed between 2005 and 2015 were retrieved from the Netherlands Cancer Registry and linked to The Nationwide Network and Registry of Histo- and Cytopathology in the Netherlands. Age-adjusted incidence rates were calculated based using the direct standardization method. Survival analyses were performed with the Kaplan–Meier method.

**Results:**

A total of 1132 patients were included for epidemiological analyses. The age-adjusted incidence rate of SB-NEN increased from 0.52 to 0.81 per 100.000 person-years between 2005 and 2015. Incidence was higher for males than females (0.93 vs. 0.69 in 2015). Most patients had grade 1 tumours (83%). Surgery was performed in 86% of patients, with resection of the primary tumour in 99%. During the study period, administration of somatostatin analogues (SSAs) increased from 5 to 22% for stage III and from 27 to 63% for stage IV disease. Mean follow-up was 61 (standard deviation 38) months. Survival data were complete for 975/1132 patients and five-year overall survival was 75% for stage I-II, 75% for stage III and 57% for stage IV.

**Conclusions:**

This study shows an increase in the incidence of SB-NEN in the Netherlands. A predominant role of surgery was found in all disease stages. Use of SSAs has increased over time.

**Supplementary Information:**

The online version contains supplementary material available at 10.1007/s00268-021-06119-y.

## Introduction

Small bowel neuroendocrine neoplasms (SB-NEN) are classified as a rare cancer type based on the incidence of <4/100.00 persons per year [1]. Despite its rarity, it represents 40% of all neoplasms of the small bowe00l [2], while simultaneously being the most common site of origin of gastroenteropancreatic neuroendocrine neoplasms (GEP-NEN) (incidence 1.05 per 100.000 person-years) [1].

Patients present with non-specific symptoms (e.g. abdominal pain) in 40% of the cases. Patients experience symptoms related to excessive hormone secretion (e.g. diarrhoea, flushing) in 20–30% of the cases [3]. Survival rates of SB-NEN are relatively high compared to other NENs, despite the delay (caused by non-specific symptoms) in diagnosis of these patients [1]. Two-thirds of the patients have locoregional disease (stage I-III) with a corresponding 5-year overall survival ranging between 97 and 100% [3, 4]. The remaining one-third has distant metastases (stage IV) with a reported 5-year overall survival of approximately 85%. This favourable outcome in the metastatic setting as compared to other malignancies might be due to the fact that some patients (with liver only metastases) are still eligible for curative intent surgery [3].

Recently, an increase in incidence and prevalence of GEP-NENs was observed in a study from the United States of America (USA) based on Surveillance, Epidemiology and End Results (SEER) data [1]. The most recent epidemiological evaluation of SB-NENs in the Netherlands was based on data between 1980 and 1997 [5]. The aim of this study was to provide an update of these Dutch data and to explore trends in epidemiology, treatment and survival outcomes of patients with grade 1 and 2 SB-NEN between 2005 and 2015.

## Methods

### Study design

All patients with grade 1 and 2 SB-NEN diagnosed between 2005 and 2015 were retrieved from the Netherlands Cancer Registry (NCR). This registry contains all cases of cancer in the Netherlands (mean total population of 16.9 million during the study period) based on hospital records, and pathology reports, treatment and survival data. Full pathology reports were requested from The Nationwide Network and Registry of Histo- and Cytopathology in the Netherlands (PALGA; *Pathologisch-Anatomisch Landelijk Geautomiseerd Archief*) [6]. This study is performed in accordance with the STROBE guidelines [7].

### Study population

Patients with pathologically proven grade 1 and 2 SB-NEN of any stage were included. The diagnosis was based on the International Classification of Disease-Oncology (ICD-O-3) morphology codes according to the World Health Organisation classification [8]. Exclusion criteria were duodenal NENs, autopsy and cytology data, benign neoplasms and non-neuroendocrine neoplasms. Neuroendocrine carcinoma, mixed adenoneuroendocrine carcinoma (MANEC) and patients with multiple primary cancers (e.g. adenocarcinoma of the colon/breast cancer/lymphoma and SB-NEN) were only used to calculate incidence rates. Patients with high-grade tumours (NET G3, neuroendocrine carcinomas and MANEC) were excluded from survival analyses. Autopsy data were excluded because those patients died of other reasons than cancer-related, and cytology data were excluded because histology is considered the standard to diagnose SB-NENs (3).

### Data collection

Primary tumour location was classified as jejunum (C17.1), ileum (C17.2) or small bowel not otherwise specified (C17.9), according to the ICD-O-3 codes. C17.9 reports were checked manually for tumour location. Tumour grade was based on the Ki67 index or mitoses index reported in the pathology reports, whichever was higher [9]. Tumour stage was reported based on the pathological tumour-node-metastasis (TNM) classification at the time of registration (6th edition during 2003–2009 and 7th edition during 2010–2016), supplemented with the clinical TNM classification [10, 11]. A one-digit summary stage (Extent of Disease) was recorded in patients without pathological confirmation of cancer [12]. The Extent of Disease code is used for patients who had no TNM stage available.

Data in both NCR and PALGA databases correspond based on unique NCR-codes. This feature was used to couple both datasets. Data regarding topography (site of primary), differentiation grade, resection margins, TNM staging, tumour positive lymph nodes reported by the NCR were cross-checked with the full pathology reports provided by PALGA. Morphology codes (cell of origin) were used in case of a mismatch in differentiation grade [13]. Data from PALGA prevailed, in case of disagreement between both datasets, because the pathology reports are more detailed. Tumour grading was based on the WHO 2010 classification. Finally, all tumours were restaged according to the 8th edition of the TNM classification to avoid differences between different TNM classifications [14]. The study period was divided into three time periods (2005–2007, 2008–2011 and 2012–2015), based on the publication date of the ENETS guidelines to compare different treatment strategies, stratified for disease stage [15, 16]. NCR only includes treatments 9 months before or after diagnosis.

In case of multiple pathology reports (e.g. one biopsy followed by resection), the first date was used for survival analyses. Time to treatment analyses could not be performed because the diagnosis was based on pathology data, which was often the date of surgery. Survival was defined as the time between date of diagnosis and date of death or censored at last follow-up date. Records of patients with pathologically proven recurrences were assessed for possible tumour dedifferentiation (i.e. tumour grade change from G1 to G2). Incidental diagnosis was defined as a patient whose first pathology report describes a resection with signs of ileus/stenosis/perforation, without previous biopsy available.

### Statistical analysis

Study populations were categorized into five age groups (<20, 20–40, 40–65, 65–80 and ≥80) according to Statistics Netherlands (CBS). Age-adjusted incidence rates were calculated, as this enables comparison with other countries, based on population data from CBS and were age-adjusted to the European Standard Population (ESP) of 2010 using the direct standardization method [17]. Baseline and treatment characteristics were compared between regional and university hospitals. Survival analyses were performed using the Kaplan–Meier method and compared with the Log-Rank test. To analyse differences in survival outcomes over the years, overall survival was calculated by stratifying for periods at which different versions of the ENETS guidelines were published (2005–2007, 2008–2011 and 2012–2015). Univariable and multivariable Cox proportional hazards regression models were used to estimate hazard ratios (HR) with 95% confidence intervals (95% CI) to identify factors associated with survival. A two-sided *P* value ≤0.05 was considered statistically significant. Data were analysed using the Statistical Package for Social Sciences (SPSS) version 26.0 (IBM Corp. Armonk, NY, USA).

## Results

**Fig.1:**
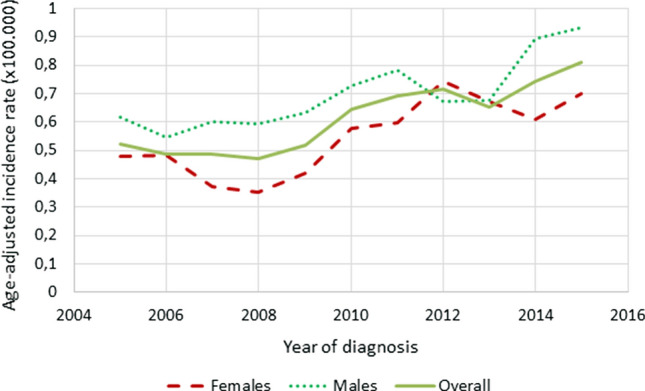
Age-adjusted incidence rates of patients diagnosed with SB-NEN between 2005 and 2015 in the Netherlands, stratified for sex

A total of 1451 patients were identified, of whom 1132 were eligible for epidemiological analysis. The age-adjusted incidence rate increased from 0.52 to 0.81 per 100.000 persons years between 2005 and 2015 (Fig. 1). Males had higher incidence rates than females throughout the years, with an incidence of 0.93 versus 0.69 per 100.000 persons in 2015.

### Patients demographics

After excluding multiple primary cancers (*N*=122), high-grade tumours (*N*=28) and MANEC (*N*=7), 975 patients were left for survival analyses. Mean age at diagnosis was 63 (SD ±12) years. Baseline characteristics are summarized in Table 1. Patients from university hospitals were significantly younger and had significantly more often multiple primary SB-NEN than patients from primary centres. All other patient and tumour characteristics were similar for the two types of hospitals. Mean follow-up was 61 (SD ±38) months, and all-cause mortality was 33%. Most patients had a grade 1 tumour (83%) (WHO 2010). Lymph node metastases (either pN1 or pN2) were present in 84% of G1 and 89% of G2 tumours (*P*=0.26). Distant metastases were more frequent in G2 (56%) than G1 (34%) tumours (*P*<0.001), and in node-positive than node-negative tumours (36% vs. 26%) (*P*=0.030).

**Table 1:** Patient and tumour characteristics stratified for centre of diagnosis

Characteristics	Missing	Total (*N*=974)	Diagnosis at:		
			Regional hospital (*N*=788)	University hospital (*N*=186)	*P* value
Sex					
Male	0	511 (52)	414 (53)	97 (52)	0.92
Age	0				
< 20		1 (0)	0 (0)	1 (1)	**0.002**
20 up to 40		26 (3)	20 (3)	6 (3)	
40 up to 65		482 (49)	372 (47)	110 (59)	
65 up to 80		386 (40)	324 (41)	62 (33)	
≥80		79 (8)	72 (9)	7 (4)	
Clinical disease stage	431 (44)		358 (83)	73 (17)	
Stage I-II		40 (4)	34 (8)	6 (5)	0.63
Stage III		119 (12)	93 (21)	26 (23)	
Stage IV		384 (39)	303 (71)	81 (72)	
Pathological TNM-stage	184 (19)		155 (84)	29 (16)	
pT					
T1		49 (5)	40 (6)	9 (6)	0.45
T2		92 (9)	79 (13)	13 (8)	
T3		386 (40)	303 (48)	83 (53)	
T4		263 (27)	211 (33)	52 (33)	
Multiple tumours	*	172 (18)	128 (17)	44 (24)	**0.031**
pN	256 (26)		214 (84)	42 (16)	
N0		109 (11)	90 (16)	19 (13)	0.71
N1		510 (52)	404 (70)	106 (74)	
N2		99 (10)	80 (14)	19 (13)	
pM1	*				
pM1a		209 (21)	168 (57)	41 (56)	0.78
pM1b		129 (13)	104 (36)	25 (34)	
pM1c		28 (3)	21 (7)	7 (10)	
Prognostic stage group	128 (13)		111 (87)	17 (13)	
Stage I-II		70 (7)	57 (8)	13 (8)	0.95
Stage III		410 (42)	327 (48)	83 (49)	
Stage IV		366 (38)	293 (43)	73 (43)	
Tumour grade	11 (1)		10 (91)	1 (9)	
Grade 1		800 (82)	652 (84)	148 (80)	0.22
Grade 2		163 (17)	126 (16)	37 (20)	
Recurrence	*				
Dedifferentiation		9/79 (11)	7/61 (12)	2/18 (11)	0.08

Bold numbers depict statistically significant *P* values

*Depicts characteristics for which missing variables could not reliably be calculated.

### Survival outcomes

**Fig.2:**
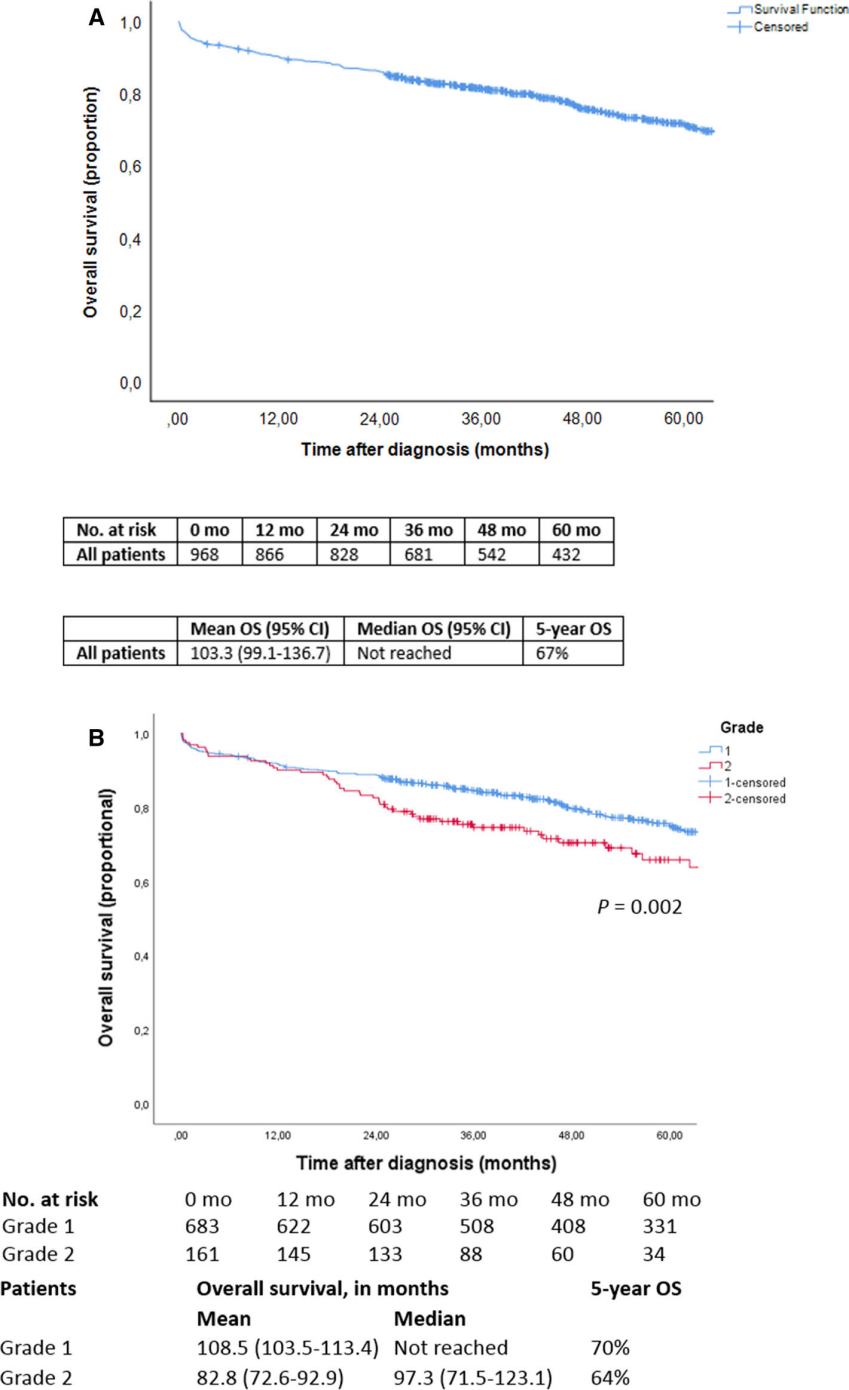

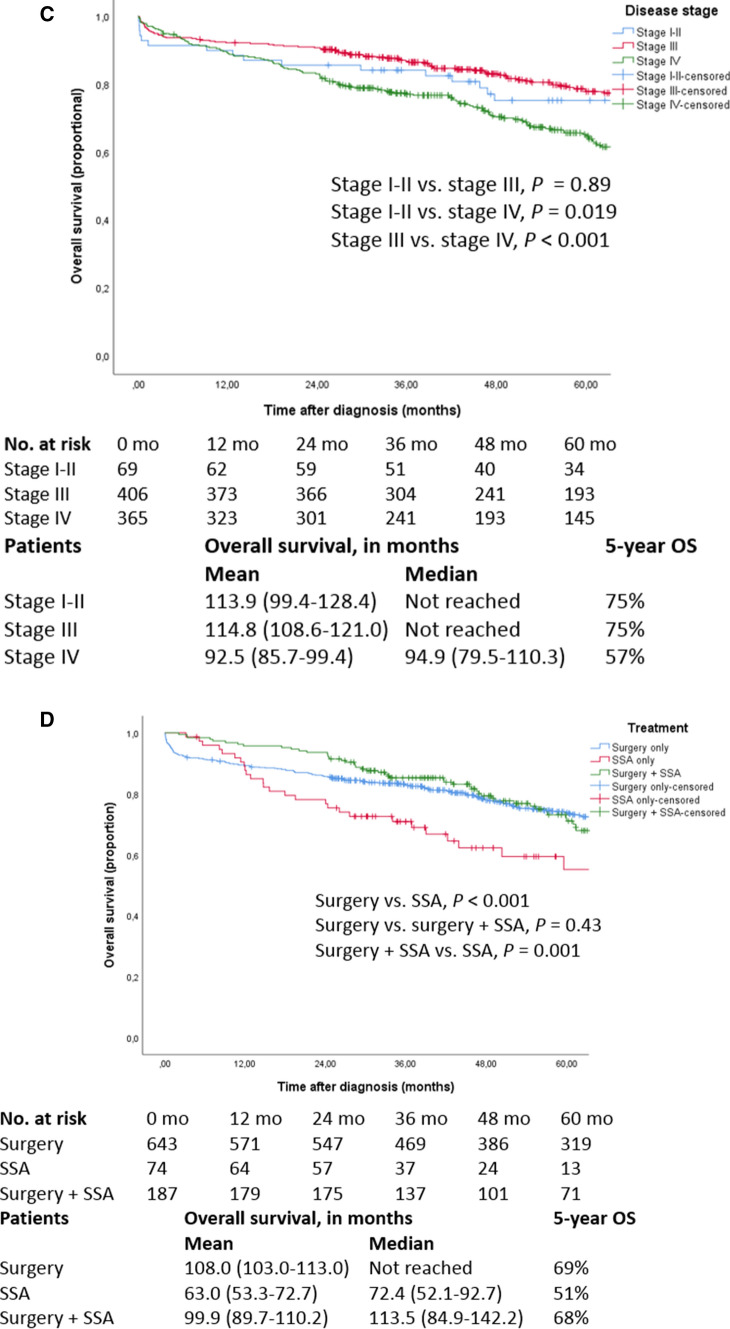
Overall survival of **(A)** all patients, **(B)** patients with different tumour grades, **(C)** patients based on tumour stage, **(D)** patients stratified for different treatments

Five-year overall survival of the entire cohort was 67% (Fig. 2a). There were no significant differences in overall survival for patients diagnosed in different years (5-year overall survival of 62% in 2005–2007, 67% in 2008–2011 and 62% in 2012–2015, *P*=0.39), or diagnosed in academic (66%) or regional hospitals (67%) (*P*=0.74). Differences in survival outcomes between different types of hospitals and stratified for disease stages were present but were not significant: stage I-II 72 versus. 89%, stage III 76 versus. 67%, stage IV 56 versus. 62% for regional vs. academic hospitals, respectively. Five-year overall survival was 70% for G1, which was significantly higher (*P*=0.002) than the 64% survival rate for G2 tumours (Fig. 2b). Stratified for stage, 5-year overall survival was 75% for stage I-II, 75% for stage III and 57% for stage IV (Fig. 2c). Stage I-II and III disease showed significantly better survival compared to stage IV disease, with an absolute difference in mean survival of at least 21 months (*P*=0.019 and *P*<0.001, respectively). Presence or absence of multifocal primary SB-NEN did not affect survival (*P*=0.75). Pathologically proven recurrence was present in 80/975 (8%) patients, and 9/80 (11%) had tumour dedifferentiation.

### Treatment strategies

The majority of the patients underwent surgery (86%), which comprised resection of the primary tumour in 99% (Table 2). The R0 resection rates increased over the years and was 84%, 81% and 62% in stage I-II, stage III and stage IV disease (Table 2). Findings that suggest an incidental diagnosis were ileus, stenosis and perforation, which were reported in 2–3% of the pathology reports. SSA use was significantly higher in university hospitals 90/223 (40%) patients, compared to 98/613 (16%) patients in regional hospitals (*P*<0.001).

**Table 2:** Trends in treatment for patients with SB-NEN in the Netherlands, according to postoperative disease stage

Stage	Treatment	2005–2007, No. (%)	2008–2011, No. (%)	2012–2015, No. (%)
Stage I-II	Total patients	13 (100)	25 (100)	32 (100)
	Primary resection^a^	13 (100)	25 (100)	32 (100)
	R0	10 (77)	20 (80)	27 (84)
	R1/2	2 (15)	2 (8)	2 (6)
	SSA	–	1 (4)	4 (13)
Stage III	Total patients	73 (100)	142 (100)	195 (100)
	Primary resection^a^	70 (96)	136 (96)	190 (97)
	R0	46 (65)	100 (74)	154 (81)
	R1/2	12 (17)	20 (15)	20 (11)
	SSA	4 (5)	17 (12)	43 (22)
	PRRT	–	1 (1)	–
	Systemic therapy	–	–	1 (1)
	No therapy	–	2 (1)	3 (2)
Stage IV	Total patients	73 (100)	123 (100)	170 (100)
	Primary resection^a^	61 (84)	89 (72)	116 (68)
	R0	31 (51)	61 (69)	72 (62)
	R1/2	15 (25)	18 (20)	24 (21)
	Metastasectomy	11 (15)	23 (19)	20 (12)
	SSA	20 (27)	54 (44)	107 (63)
	Systemic therapy	2 (3)	2 (2)	2 (1)
	RFA	1 (1)	1 (1)	–
	PRRT	1 (1)	2 (2)	–
	Embolization	–	2 (2)	3 (2)
	Radiotherapy	–	1 (1)	–
	No therapy	6 (8)	2 (2)	9 (5)

Treatments are reported in a range of 9 months before or after diagnosis

^a^Resection margins do not add up to 100% due to missing variables

*PRRT* peptide receptor radioligand therapy, *RFA* radiofrequency ablation, *SSA* somatostatin analogues

Survival was not different for stage IV disease after primary or simultaneous resection of primary and metastases (Supplementary Fig. 1). Survival after surgery and after surgery combined with somatostatin analogues (SSAs) were significantly longer than survival after SSA alone (*P*<0.001, *P*=0.001) (Fig. 2d). A similar effect is observed in the presence of distant metastases (Supplementary Fig. 2). Surgery, SSA or a combination of both were included in the survival analyses, since the other treatment groups were too small.

In Table 2, time trends in treatment modalities for SB-NEN are presented. All patients with stage I-II disease underwent resection. Throughout the years, the resection rate for stage I-III disease remained high (96–100%) and administration of SSAs increased from 5 to 22% for stage III between 2005 and 2015. In patients with stage IV disease, the primary tumour resection rate decreased, while administration of SSAs more than doubled during the study period from 27 to 63%. Complete (R0) resections were performed in 57/62 (90%) patients with stage I-II disease, 300/352 (85%) patients with stage III disease and 164/221 (74%) patients with stage IV disease. All treatments that are reported in the NCR database took place within 9 months from diagnosis.

### Factors associated with survival

Male sex, age between 20 and 40, 65–80 and ≥80 years, stage I-II and III disease, grade 2 tumours, surgery and SSA use showed an association with a shorter overall survival in univariable analysis. In multivariable analysis, male sex (HR 1.39, 95% CI 1.09–1.78, *P*=0.008), age between 65 and 80 years (HR 2.93, 95% CI 2.23–3.87, *P*<0.001), age ≥80 years (HR 9.99, 95% CI 6.61–15.11, *P*<0.001), stage III disease (HR 0.51, 95% CI 0.38–0.69, *P*<0.001 with stage IV as reference), grade 2 tumours (HR 1.48, 95% CI 1.09–2.02, *P*=0.013) and not having surgery (HR 1.50, 95% CI 1.07–2.09, *P*=0.018) all showed a significant association with a shorter overall survival. The results of univariable and multivariable analyses for overall survival are shown in Table 3.

**Table 3:** Univariable and multivariable survival analyses of patients with SB-NEN in the Netherlands

Risk factors	Univariable analysis	Multivariable analysis		
	HR (95% CI)	*P* value	HR (95% CI)	*P* value
Sex				
Male	1.19 (0.96–1.48)	**0.12**	1.39 (1.09–1.78)	**0.008**
Female	1 [Reference]	1 [Reference]		
Age				
<20	–	–		
20 up to 40	0.20 (0.03–1.41)	**0.11**	0.27 (0.04–1.95)	0.19
40 up to 65	1 [Reference]	1 [Reference]		
65 up to 80	2.81 (2.19–3.60)	**<0.001**	2.93 (2.23–3.87)	**<0.001**
≥80	6.34 (4.52–8.89)	**<0.001**	9.99 (6.61–15.11)	**<0.001**
Multiple primary SB-NEN				
Yes	1.03 (0.77–1.38)	0.83	–	
No	1 [Reference]	–		
Disease stage				
Stage I-II	0.56 (0.34–0.91)	**0.020**	0.63 (0.37–1.07)	0.08
Stage III	0.55 (0.42–0.70)	**<0.001**	0.51 (0.38–0.69)	**<0.001**
Stage IV	1 [Reference]			
Tumour grade				
Grade 1	1 [Reference]		1 [Reference]	
Grade 2	1.49 (1.12–1.99)	**0.006**	1.48 (1.09–2.02)	**0.013**
Resection margin				
R0	1 [Reference]		–	
R1/2	1.14 (0.81–1.60)	0.44	–	
Surgery				
Yes	1 [Reference]	1 [Reference]		
No	1.99 (1.53–2.62)	**<0.001**	1.50 (1.07–2.09)	**0.018**
SSA				
Yes	1.25 (0.98–1.60)	**0.07**	1.09 (0.81–1.46)	0.57
No	1 [Reference]	1 [Reference]		

Bold numbers depict statistically significant *P* values

## Discussion

This population-based study observed an increase in incidence of grade 1 and 2 SB-NEN between 2005 and 2015, and surgery remained the mainstay of treatment. The most remarkable changes were seen for stage IV SB-NEN, with a reduced rate of surgery and substantial increase in the use of SSAs. Survival did not change over time. Five-year overall survival rate of 75% for stage I-II disease was relatively low and similar to survival of patients with stage III SB-NEN.

The increase in incidence of SB-NEN in the Netherlands (56% in the last 10 years (0.81 per 100.000 persons) can be explained by more clinical awareness and increased utilization of cross-sectional imaging for any reason, even including screening. Such imaging might reveal asymptomatic liver lesions or lymph node metastases in the mesentery, that eventually turn-out to originate from SB-NEN. Furthermore, SB-NEN might increasingly be diagnosed as incidentalomas by pathologists in resection specimens after surgery for other diagnoses. The incidence has also risen compared to the prior study conducted in the Netherlands, which dates from 2001 [5]. Previous population-based studies conducted in Europe reported comparable increased incidence rates: 0.29 in Austria (2004–2005, grade 1 and 2 only), 0.30 in Italy (1981–2005, grades not reported), 0.80 in Iceland (2000–2014, grades not reported) and 0.81 in Norway (1993–2004, grades not reported), per 100.000 persons (18–21). Another explanation for the increase in incidence is an ageing population.

While some studies report relatively high 5-year overall survival rates for stage IV disease (69–85%), this trend is not seen in the Netherlands where survival rates are lower (57%) [1–22]. Similarly, 5-year overall survival in stage I-II was relatively low (75%) compared to the literature [3]. This could be due to differences in patient populations regarding competing risks of death, besides pathological classification, inclusion criteria (because only grade 1 and 2 were included), statistical methods, treatment differences between countries, and different inclusion periods. Survival outcomes differ between treatment strategies (Fig. 2d), but this is probably due to the imbalance in disease stages among the different treatment modalities (Table 2). We also observed a relatively low pathologically proven recurrence rate (8%), which is most likely an underestimation, as other studies report recurrence rates as high as 31–64% [23, 24]. The low recurrence rate might be explained by the high frequency of lack of histological confirmation of recurrent disease and subsequently under-reporting in the PALGA database. Indeed, Cives et al. diagnosed macroscopic recurrence by imaging or surgical exploration, and Le Roux et al. diagnosed recurrence in asymptomatic patients with imaging during follow-up monitoring [23, 24].

Multifocal SB-NEN were only present in 18% of the patients, which differs from the literature (45–54%) [25, 26]. However, our data show that tumour multifocality is not associated with overall survival [26]. A Swiss population-based study investigating treatment sequences in NENs found that 80% of SB-NEN patients received surgery (either with or without subsequent therapy), which is a similar rate as found by the present study (86%) [27]. An increase in SSA administration was seen for all stages, with a doubling for stage IV SB-NEN. This is probably a consequence of the positive effects of SSAs that have been reported: reduction of excessive hormone secretion by (liver) metastases, prolonged progression-free survival and anti-proliferative effects [28, 29].

Surprisingly, no significant differences in neither clinical or pathological TNM stages were observed between university and regional hospitals. Hence, patients were not referred for surgical resection to either one of those centres based on cTNM stages between 2005 and 2015. It is likely that centralization improves patient outcomes as choosing the right treatment strategy is evenly, if not more, challenging than executing the treatment itself (except for complex surgery). Nevertheless, current data did not show any survival difference between academic and regional hospitals. Probably, clinicians should focus first on discussing all patients in a multidisciplinary team (MDT) meeting in a specialized centre for NENs. Taken together, an international, multicentre registry with data on patient level is needed to carefully investigate diagnostic, treatment and outcome variables.

Long-term nationwide population-based data were used for this study, making it more representative than cohort studies and enable description of trends over the years. However, the findings of this study should be seen in light of some limitations. Comorbidity data were missing which might have influenced survival. To reduce this effect, patients with multiple primary cancers were excluded from survival analyses. Second, pTNM stage was used as a stratifying factor although in real-life treatment strategies are chosen based on cTNM stage. Third, pathological classification of NENs according to the WHO has changed over the years. This could lead to wrongly classified lesions. Fourth, imaging data during follow-up were not present, which is especially useful to give insights in disease recurrence and therefore the actual incidence is probably higher than reported by the NCR. Finally, treatment is only registered within 9 months from diagnosis, and the dataset lacks details on several specific local treatments of metastatic disease, such as peptide receptor radionuclide therapy, embolization, stereotactic radiotherapy and thermal ablation. This limits evaluation of all types of treatments given during complete follow-up.

## Conclusions

In conclusion, this study showed an increase in the incidence of grade 1 and 2 SB-NEN, which is not uniformly reported in Europe. Surgery is still the cornerstone of treatment. An increase in use of SSAs was observed in stage IV disease over time. Stage-dependent survival was relatively low compared to the literature and remained similar over time.

## Supplementary Information

Below is the link to the electronic supplementary material.

Supplementary file1 (TIF 123 kb)

Supplementary file2 (TIF 96 kb)
