# Supplementary figures and images for: Update on Incidence, Prevalence, Treatment and Survival of Patients with Small Bowel Neuroendocrine Neoplasms in the Netherlands
Source: World J Surg. 2021 Apr 24;45(8):2482–91. doi: 10.1007/s00268-021-06119-y (PMC8236032; doi:10.1007/s00268-021-06119-y)

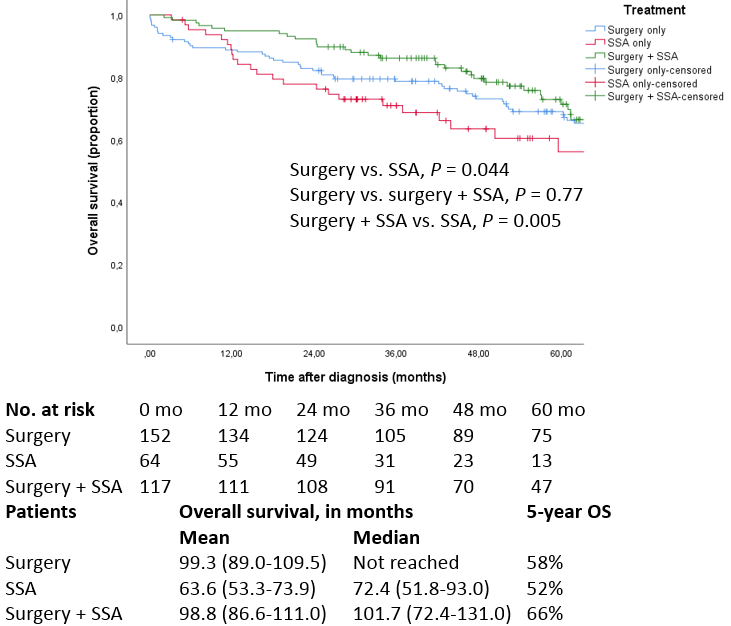

Supplement: Supplementary file 1 — Supplementary file1 (TIF 123 kb) [file 268_2021_6119_MOESM1_ESM.tif]

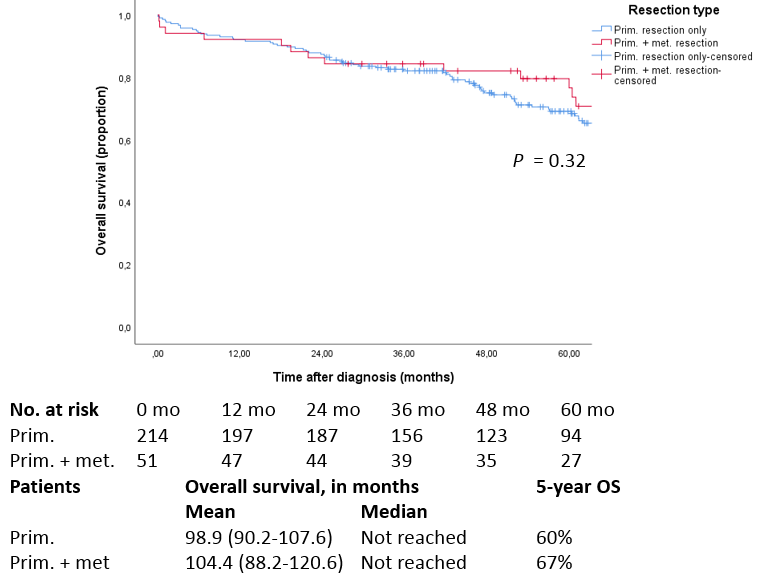

Supplement: Supplementary file 2 — Supplementary file2 (TIF 96 kb) [file 268_2021_6119_MOESM2_ESM.tif]
